# Formulation Effects in the Antioxidant Activity of Extract from the Leaves of *Cymbopogon citratus* (DC) Stapf

**DOI:** 10.3390/molecules26154518

**Published:** 2021-07-27

**Authors:** Raquel Sousa, Artur Figueirinha, Maria Teresa Batista, Maria Eugénia Pina

**Affiliations:** 1 Faculty of Pharmacy, University of Coimbra, Pólo das Ciências da Saúde, Azinhaga de Santa Comba, 3000-548 Coimbra, Portugal; rakel.sgs@gmail.com (R.S.); amfigueirinha@ff.uc.pt (A.F.); mtpmb@ff.uc.pt (M.T.B.); 2 LAQV, REQUIMTE, Faculty of Pharmacy, University of Coimbra, Pólo das Ciências da Saúde, Azinhaga de Santa Comba, 3000-548 Coimbra, Portugal; 3 CIEPQPF, FFUC, University of Coimbra, Pólo das Ciências da Saúde, Azinhaga de Santa Comba, 3000-548 Coimbra, Portugal

**Keywords:** *Cymbopogon citratus*, gelatine capsules, biological activity, chemical stability

## Abstract

*Cymbopogon citratus* DC (Stapf.) is a perennial grass and it is distributed around the world. It is used as a condiment for food and beverage flavouring in the form of infusions and decoctions of its dried leaves. Our previous studies have shown antioxidant, anti-inflammatory and gastroprotective activities for the infusion and its phenolic fractions. The aim of the present work was to develop oral dosage forms from a *Cymbopogon citratus* extract to be used as a functional food with antioxidant properties. Initially, an essential oil-free infusion was prepared, lyophilized and characterized by HPLC-PDA. Total phenols were quantified with the Folin–Ciocalteu method and the antioxidant activity was assessed by DPPH assay. Gelatine capsules containing the extract with different excipients, selected after DSC and IR trials, were prepared. A formulation exhibiting better antioxidant behaviour in a gastric environment was attained. These results suggest that the proposed formulation for this extract could be a valuable antioxidant product and, consequently, make an important contribution to “preventing” and minimizing diseases related to oxidative stress conditions.

## 1. Introduction

*Cymbopogon citratus* (known as lemongrass) belongs to the family Poaceae and is one of the 30 species comprising the *Cymbopogon* genus [1]. The plant is native to Southeast Asia and develops in tropical and subtropical regions [2]. In traditional medicine fresh or dried leaf infusions are usually prepared [1]. They are used, for example, in the treatment of gastrointestinal and nervous disturbances, and also as anti-inflammatory, analgesic, sedative, antipyretic, antioxidant, and for metabolic diseases (such as diabetes) [3]. Scientific research has identified various properties associated with *Cymbopogon citratus* leaves, such as antimicrobial, antiprotozoal, anti-rheumatic, antitumoural, cardio-protective, anti-inflammatory and gastroprotective properties [4,5,6,7,8]. Additionally, *Cymbopogon citratus* is used as a flavouring; in perfumery, cosmetics and aromatherapy; and as an insecticide [3]. The phytochemicals in *Cymbopogon citratus* leaves are phenolic compounds; specifically, hydroxycinnamic acids (such as chlorogenic, caffeic and *p*-coumaric acid derivatives), flavones (luteolin and apigenin derivatives), condensed-type tannins [9,10], triterpenes, electrolytes and minerals [3,11,12]. Citral is the main component of its essential oil [1,13]. The infusions, and their phenolic fractions, have been shown to demonstrate anti-inflammatory, antioxidant and antiradical activities [7,10,14].

Reactive oxygen species (ROS), when present in high quantities and in a sustained manner, lead the body to an oxidative stress situation, causing cellular damage that is reflected in multiple disorders, such as heart and neurodegenerative diseases, cancer and general inflammation. The ingestion of exogenous antioxidants helps to minimize such damages. Methanol and methanol/water extracts, infusions and decoctions of *Cymbopogon citratus*, and particularly the phenolic fractions, have been shown to have the ability to eliminate ROS in several trials of antioxidant activity [10,15,16]. It has been proposed that the antioxidant activity of polyphenol fractions of the plant may have a key role in endothelial dysfunctions associated with oxidative stress, as it decreases ROS production in human umbilical vein endothelial cells (HUVECs) and decreases the vasoconstriction induced by thromboxane A2 [17]. Another therapeutic application is in atherosclerosis, after *C*-glycosylflavonoids have decreased the oxidation of low-density lipoproteins [18]. Studies performed by Tiwari et al. [19] showed increases of superoxide dismutase activity and glutathione content and reductions of ROS production and lipid peroxidation in mice alveolar macrophages. The antioxidant activity has been attributed to flavonoids, tannins [10] and vitamins contents [12]. Despite these activities, many of these phytochemicals can show different behaviour in vivo due to their stability in gastric media. To overcome this, a formulation of the extract might enhance its activity.

The capsule formulation offers the advantage of requiring fewer excipients than other oral dosage forms, in addition to its greater flexibility. Due to their faster disintegration and dissolution, capsules also provide faster drug release; additionally, the compliance and convenience are improved [20]. Therefore, with the growing interest in the use of alternatives that can prevent or minimize harmful oxidative stress effects, this study aimed to develop gelatine capsules for oral administration from an antioxidant *Cymbopogon citratus* extract, based on assessment of polyphenol composition and antioxidant activity variations in vitro, by simulating the gastric conditions.

## 2. Results 

### 2.1. Characterization of Cymbopogon Citratus Extract

Evaluation of water content, antioxidant activity and total phenols, along with determination of polyphenols by HPLC-PDA, were used to characterize the extract. These tests had as their main objective the definition of extract features to their respective standardization.

#### 2.1.1. Determination of Water Content

Extract water content was determined as described in Section 4.4. The obtained value, 0.20% water content, is in accordance with the limits established in the European Pharmacopoeia [21] for a dry extract.

#### 2.1.2. Identification of Polyphenols by HPLC-PDA

**Figure 1:**
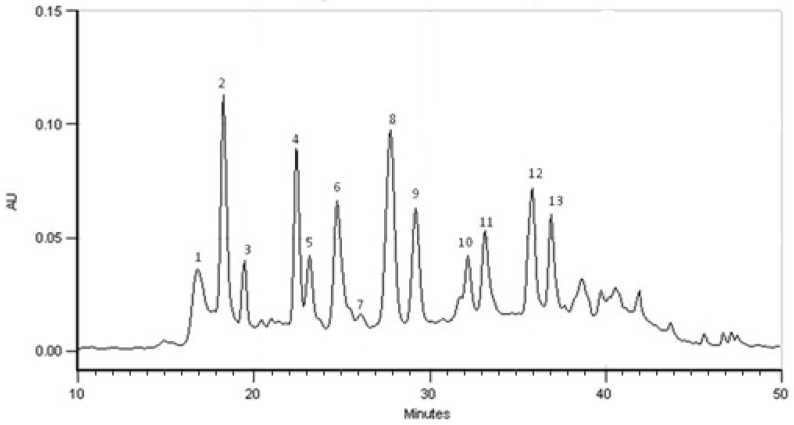
Chromatographic profile obtained from *Cymbopogon citratus* extract at 280 nm. Identification of the compounds is provided in Table 1.

Figure 1 represents the chromatographic profile obtained by HPLC-PDA, at 280 nm, from the extract at a concentration of 1.8 mg/mL in methanol–water. UV spectra show the presence of tannins (1); phenolic acids, namely, caffeic, ferulic and *p*-coumaric acids (2–4); and flavonoids, mainly flavones such as luteolin and apigenin derivatives (5–13).

Table 1 summarizes the phenolic compounds identified in the extract by comparing the UV spectra and retention times in the present research with those previously identified by the group from the Laboratory of Pharmacognosy, University of Coimbra, using HPLC-PDA-ESI/MS^n^ [10,14]. Among the flavonoids, *C*- and *O*-glycosides were identified, mostly *C*-glycosylated derivatives (Table 1).

#### 2.1.3. Antioxidant Activity and Total Phenols Evaluation

Extract antioxidant activity was calculated by DPPH test and expressed as EC_50_ values using the equation y = 1.3212x − 1.1863, with a correlation coefficient of 0.9986. The EC_50_ value found, 38.75 ± 1.09 µg/mL, was similar to the value of 41.72 ± 0.05 µg/mL cited by Tavares et al. [22].

Total phenols content assessed by the Folin–Ciocalteu method was 70.20 ± 0.02 mg equivalents of gallic acid per gram of extract. This value was obtained through the calibration curve y = 0.1081x − 0.0109, R^2^ = 0.9904. It is one of the highest obtained from this material, being in line with the results of Costa et al. [23], where it was concluded that in summer the total phenol content is at its highest.

#### 2.1.4. Detection of *n*-Hexane Traces in the Extract

*n*-Hexane traces in the extract were monitored by GC with headspace. No trace of the solvent was verified, confirming the safety of this extract for human use.

#### 2.1.5. Differential Scanning Calorimetry

**Figure 2:**
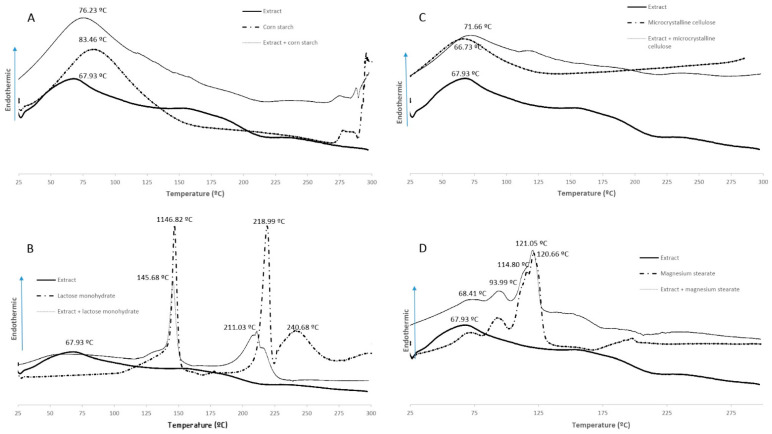
DSC thermograms of the extract, excipients and extract—excipients mixtures (1:1): (**A**) corn starch; (**B**) lactose monohydrate; (**C**) microcrystalline cellulose; (**D**) magnesium stearate.

The results obtained from the DSC studies of the extract, excipients and extract–excipient mixtures are presented in Figure 2 and do not show evidence of important interactions.

#### 2.1.6. Infrared Spectroscopy

**Figure 3:**
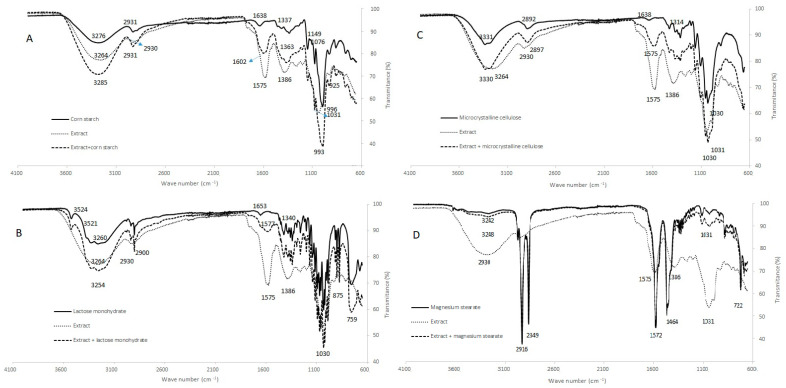
FTIR-ATR spectra of the extract, excipients and extract—excipient mixtures (1:1): (**A**) corn starch; (**B**) lactose monohydrate; (**C**) microcrystalline cellulose; (**D**) magnesium stearate.

FTIR-ATR spectra of the extract, excipients and extract–excipient mixtures are provided in Figure 3 and do not show worrying interactions.

### 2.2. Oral Dosage Forms Development

The formulations developed and studied in this work can be found in Section 4.10. Gelatin capsules were prepared with 330 mg of extract, a mass based on the extractive yield obtained from the amount of the plant used in traditional medicine (2 g of dried leaves). No liver toxicity was verified in male Wistar rats for extract dosages of 34 and 68 mg/Kg of body weight [7].

Attending to the extract density (0.879 g/mL), the busy volume in the gelatinous receptacle was 0.375 mL and, consequently, capsules of the number 0 were selected.

#### 2.2.1. Mass Uniformity 

All formulations presented masses within the range defined by the European Pharmacopoeia [21]. The obtained results for the average, minimum and maximum weights were, respectively, 483.30 (487.55, 477.71; CV = 0.94%), 529.95 (528.36, 531.96; CV 0.25%), and 483.20 mg (477.30, 486.50; CV 0.67%) for the formulations of extract with the corn starch (F1) and lactose monohydrate (F2) excipients and for the mixture of all tested excipients (F3).

#### 2.2.2. Content Uniformity

This parameter was evaluated by quantification of total phenols using the Folin–Ciocalteu method. A blank control test was performed with the excipients in the amounts present in formulations. The content uniformity, expressed in mg of gallic acid per 330 mg of the extract, is illustrated in Table 2. The formulations respected the limits fixed in the European Pharmacopoeia [21] (85–115% of the average content).

#### 2.2.3. Dissolution Tests

The results obtained to validate the dissolution test in artificial gastric juice were as follows: specificity—no interference of excipients; linearity—Y intercept 0, correlation coefficient = 1; accuracy—per cent recovery 98–101.26%; repeatability—CV = 4.49%.

**Figure 4:**
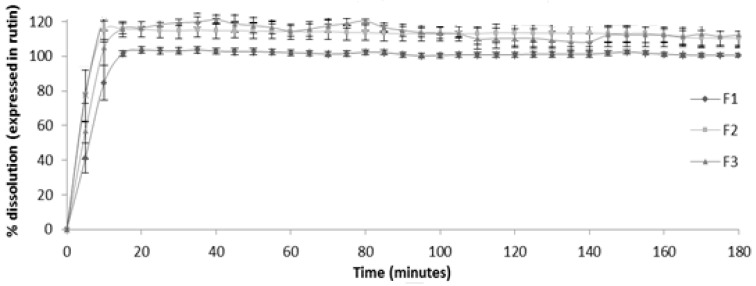
Dissolution profiles for the different developed formulations. F1—extract and corn starch; F2—extract and lactose monohydrate; F3—extract and corn starch, lactose monohydrate, magnesium stearate and microcrystalline cellulose.

Tested formulations were totally dissolved after 15 min (Figure 4). These dissolution values were in accordance with those defined officially for the conventional release dosage forms, more specifically, very quick release dosage forms [24].

Dissolution profiles showed that two formulations (F2 and F3) presented dissolution values above 100%. Corn starch was the excipient that conferred a dissolution value around 100% (formulation F1). On the other hand, the interactions verified with lactose monohydrate or with the mixture of the excipients (corn starch, lactose monohydrate, magnesium stearate and microcrystalline cellulose) justify these results.

#### 2.2.4. Selection of Formulation

Based on pre-formulation development and quality control tests (dissolution profile around 100% and compatibility between extract/excipient), the formulation F1 (extract and corn starch) was selected and evaluated for stability in gastric juice and antioxidant activity.

### 2.3. Evaluation of the Formulations after Gastric Juice Action 

#### 2.3.1. Assessing of Phenolic Compounds Stability in Gastric Juice by HPLC-PDA

**Figure 5:**
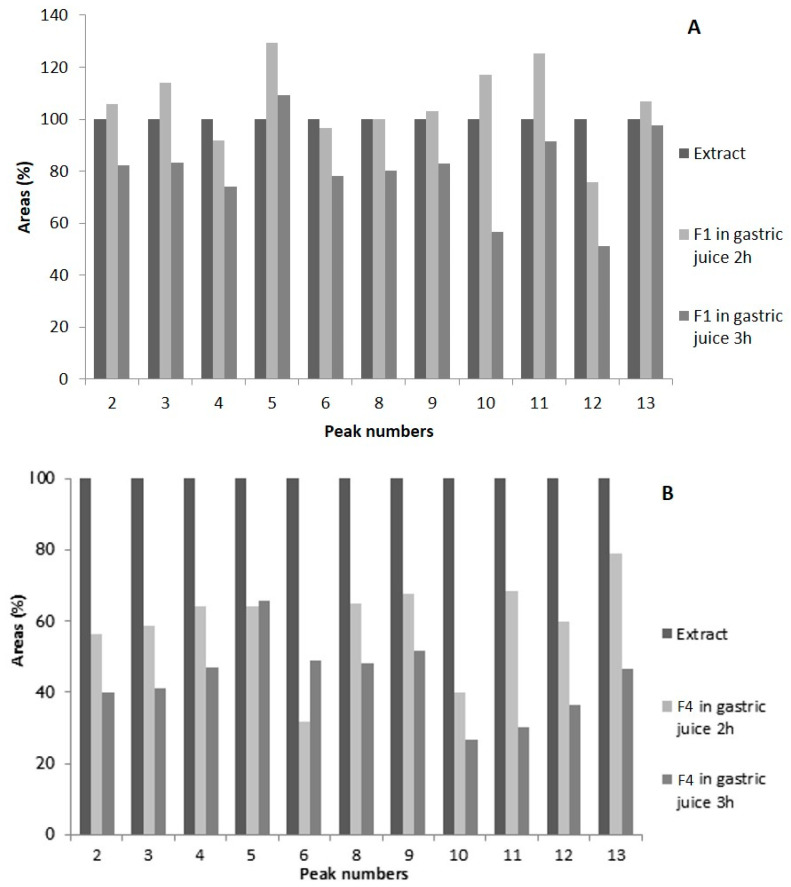
Representation of the area variation (at 320 nm), in per cent, of the peaks obtained (2–13) by HPLC-PDA for the F1 and F4 formulations in relation to the extract not subjected to gastric juice. F1—Extract and corn starch (**A**); F4—extract (**B**). Average values calculated from *n* = 2. Identification of peaks in Table 1.

The stability of the formulations containing the extract with corn starch (F1) and only the extract (F4) was verified by HPLC-PDA after 2 and 3 h of contact with the simulated gastric juice (Figure 5A,B, respectively).

Overall there was a decrease in the phenolic compounds content, mainly at 3 h of the assay (Figure 5), as well as the appearance of new compounds. At the retention time of 17.5 min, a new compound occurred, presenting a UV spectrum profile and wavelengths (278 nm) typical of flavans. The increase of these compounds and the decrease of the baseline (the rise of which is related to the tannins) seem to indicate acid degradation of existing tannins in the extract, since *Cymbopogon citratus* presents in its composition procyanidins of type B, catechin and derivatives of luteoliflavan and apigeniflavan [9].

The formulation of the extract with corn starch (F1) showed a decrease in the area of the peaks related to the phenolic compounds (Figure 5A), mainly at 3 h of the assay; however, it was less marked than the formulation containing only the extract (F4) (Figure 5B). This result may indicate the corn starch protection of the compounds, which prevented their degradation. At 2 h, we observed a decrease for the area of co-eluted compounds in peak 12 (6-*C*-pentosyl luteolin and 2″-*O*-α-*L*-rhamnosyl-6-*C-*α-arabinofuranosyl luteolin). However, peaks 5 (6-*C*-β-glucopyranosyl-8-*C*-α-arabinopyranosyl luteolin), 10 (7-*O*-β-glucopyranosyl luteolin) and 11 (7-*O*-neohesperosyl luteolin and 6-*C*-pentosyl-8-*C*-deoxyhexosyl luteolin) demonstrated an increase (Figure 5A). Similar behaviour was observed for some olive pomace polyphenols in a formulation with cyclodextrins [25]. At 3h of contact with gastric juice, the reduction of the area of the peaks was verified for most of the compounds but was higher for the F4 formulation compounds (Figure 5B). 

#### 2.3.2. Evaluation of Antioxidant Activity by DPPH Assay after Gastric Juice Action

**Figure 6:**
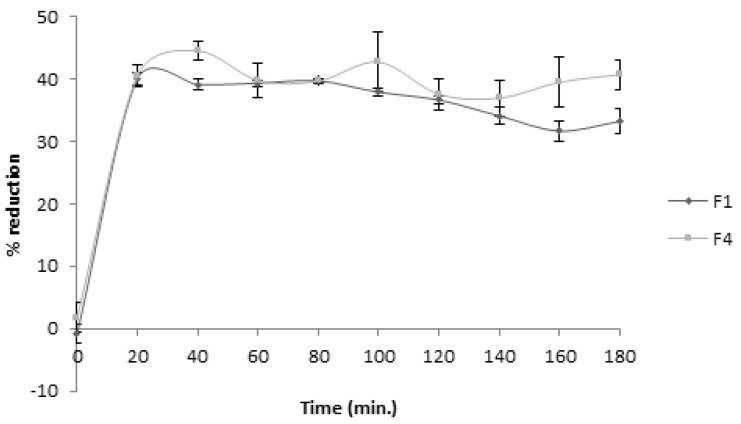
Antioxidant activity evaluation of the formulations after gastric juice action.

In order to evaluate the extract antioxidant stability after its formulation, the DPPH assay was performed by using aliquots of the reaction medium, collected every 20 min for 3 h. The obtained results for the formulation with the extract and corn starch (F1) (Figure 6) indicated a decrease, neither too sharp nor significant, of the capacity to reduce the DPPH with the time. The antioxidant capacity remained roughly similar up to about 80 min, with a slight decrease occurring afterwards up to 180 min. For the capsule containing only the extract (formulation F4), some fluctuations in the percentage of reduction were verified. Comparing these results with the value which would be expected for this concentration, according to the EC_50_ value (31.87%), the activity slightly increased after gastric juice action. 

Although the F4 formulation was found to be slightly more active toward the DPPH radical than F1, the results obtained suggest that the latter is more stable and can confer greater bioaccessibility of phenolic compounds along the gastrointestinal tract. 

## 3. Discussion

Essential oil-free infusions from *Cymbopogon citratus* leaves and their phenolic fractions containing phenolic acids, flavonoids or tannins have been studied by us previously with regard to their antioxidant, anti-inflammatory and gastroprotective activities, to which phenolic compounds were proven to contribute [7,8,10]. Flavonoids and tannins show significant anti-inflammatory activity, with *C*-glycosylflavones corresponding to the predominant flavonoids of the extract [26]. Recent studies have highlighted the beneficial effects of *C*-glycosylflavonoids on human health, namely antioxidant, anti-inflammatory, anticancer and anti-diabetic effects [27,28,29], to which the capacity to scavenge for reactive oxygen species contributes. In fact, the overproduction of ROS has been related to the development of chronic and degenerative diseases, specifically those affecting the gastrointestinal tract, such as bowel disease, and many other ailments, such as the diabetes mellitus, cardiovascular diseases and various types of cancer, for instance, cancer of the colon.

The excipients used in the formulation were chosen by the pre-formulation assays in the DSC and FTIR-ATR studies. Of the four excipients, only lactose monohydrate showed a possible interaction with the extract (Figure 2); the lactose curve demonstrated three endothermic events related to dehydration (146.82 °C), melting (218.99 °C) and thermal decomposition (240.86 °C), respectively, which were identical to the results obtained by Pereira et al. [30] and may suggest chemical incompatibility [31].

The analyses by FTIR-ATR did not show evidence of interactions, except for the case of the mixture of the extract with lactose monohydrate (Figure 3B), which exhibited a spectrum corresponding to the sum of the spectra of the isolated compounds, with the exception of the band at 1575 cm^−1^. This appeared to be greatly reduced in the mixture compared to what would be expected, thus confirming the study carried out by DSC. 

Dissolution profiles showed that two formulations (F2 and F3) presented dissolution values above 100%. The interaction was verified with lactose monohydrate and with the mixture of the excipients (corn starch, lactose monohydrate, magnesium stearate and microcrystalline cellulose), justifying these results. The findings suggest that the formulated herbal capsules of *Cymbopogon citratus* extract possess characteristics within the permitted range for conventional dosage forms specified by pharmacopoeial standards. Identical results were obtained by Esmaeili et al. [32] in their study about the formulation and evaluation of oral hard gelatine capsules from *Pinus eldarica* bark extract, which also contains many polyphenolic compounds.

In the presence of corn starch, the extract showed greater stability in the phenolic compounds. This result suggests that the corn starch protection prevented their degradation. Several studies have reported the ability of corn starch to form complexes with ligands, such as iodine, linear alcohols, lipids or ibuprofen, through the conformation change of the amylose, which forms a helix structure and allows the creation of a hydrophobic cavity where these ligands can be accommodated. In the case of ibuprofen, it has been observed that the complex formed with starch is stable in gastric juice during its degradation in the small intestine due to action of amylase [33,34]. This ability of the starch to form stable complexes in gastric conditions may explain the minor degradation of the phenolic compounds in the presence of this excipient, which may not occur similarly in all present compounds in the extract. An in vitro digestion study by Ferruzzi et al. [35] showed that a lower degradation of phenolic compounds occurred in the presence of the starch, the stability being related to the phenolic compound structure. In general, the stability of phenolic compounds in gastric juice appears to be related to the type of glycosylation. In fact, the detected diminution was more pronounced for the *O*-glycosides, which could be explained by their susceptibility to acid hydrolysis. Concerning *C*-glycosides, they most likely remain almost intact at the time of contact with gastric juice. The degradation observed in the flavonoids is in agreement with the findings of the study undertaken by Ahmad-Qasem et al. [36] on olive leaves, which contain luteolin and apigenin derivatives. In this study, the authors demonstrated a reduction of phenols in the gastric phase due to pH and enzymatic activity; however, luteolin 7-*O*-glucoside was the less stable component throughout the digestion. 

In the case of phenolic acids, complexation with starch has been verified, depending of its structure [37]. These researchers verified that this property contributes more positively to caffeic acid stability than to ferulic acid. In our study, a lower degradation for the caffeic acid derivatives was also observed. 

The degradation of phenolic compounds in the formulation containing only the extract (F4) led to an increase in its antioxidant activity (Figure 6). This could have resulted from the conversion of glycosides to aglycones, which demonstrate greater antioxidant activity. On the other hand, glycosides can show other interesting activities, such as local enzymatic inhibition effects if they manage to remain intact in the gastric environment. As a consequence, depending on the excipient used, greater or lesser degradation of phenols can be obtained, thus resulting in different biological effects.

## 4. Materials and Methods

### 4.1. Chemical Materials

2,2-Diphenyl-1-picrylhydrazyl (DPPH) and rutin were purchased from Sigma-Aldrich (St. Louis, MO, USA). Glacial acetic acid, 98–100% formic acid, anhydrous sodium acetate, acetone, sodium carbonate, absolute ethanol, methanol (HPLC grade), *n*-hexane, pepsin and the Folin–Ciocalteu reagent were purchased from Merck (Lisbon, Portugal). Hydrochloric acid (37%) and lactose monohydrate were obtained from Scharlau (Barcelona, Spain). Corn starch and microcrystalline cellulose were acquired from Labor Spirit Ltd. (Loures, Portugal). Gallic acid was obtained from Fluka (Steinheim, Switzerland); sodium chloride and magnesium stearate were purchased from Panreac (Prior Velho, Portugal).

### 4.2. Botanical Material

Dried leaves of *Cymbopogon citratus* (DC) Stapf., were acquired from ERVITAL (Mezio, Castro Daire, Portugal). The plant was collected in July 2011 and its identity was confirmed by J. Paiva (Department of Life Sciences, University of Coimbra, Coimbra, Portugal).

### 4.3. Preparation of Cymbopogon Citratus Extract 

A *Cymbopogon citratus* infusion was prepared according to Figueirinha et al. [10]. Boiling water (150 mL) was added to 5 g of plant leaves previously powdered and sieved (60Mesh). The infusion was left to rest for 15 min and then filtered under vacuum. An extraction with *n*-hexane (three times in the ratio 1:1) was performed to remove the lipophilic compounds. The aqueous fraction was then concentrated on a rotary evaporator (Büchi) and subsequently lyophilized and stored at −20 °C until use. The extractive yield was 18.4 g/100 g of dry plant.

### 4.4. Determination of Water Content

The water content of the dried leaves and of the lyophilized extract (designated by extract in this work) was determined in triplicate through the loss of water per 0.5 g sample dried at 100 °C for at least 24 h, keeping warm up to the constant weight. The water content was determined in triplicate with the following equation:

(1) $$\%\;\mathrm{humidity}\;=\frac{\left(\mathrm{initial}\;\mathrm{mass}-\mathrm{mass}\;\mathrm{after}\;\mathrm{drying}\right)}{\mathrm{initial}\;\mathrm{mass}}\times 100$$

### 4.5. High Performance Liquid Chromatography

HPLC analyses were performed on a high-resolution liquid chromatograph (HPLC) connected to a photodiode-array detector (PDA) (Gilson^®^ Electronics SA, Villiers le Bel, France). A Waters^®^ RP18 Spherisorb ODS-2 column (4.6 × 250 mm) with particles size of 5 µm, set at 24 °C, and a C18 pre-column KS 30/4 Nucleosil 5 µm (Macherey-Nagel) were used. A mobile phase of 5% (*v*/*v*) aqueous formic acid (A) and methanol (B) was used, at a flow rate of 1 mL/min, with a discontinuous gradient: 5–15% B (0–10 min), 15–30% B (10–15 min), 30–35% B (15–25 min), 35–50% B (25–35 min), 50–80% B (35–40 min). This was followed by an isocratic elution for 20 min. An injection volume of 100 µL was used for the samples prepared with 1.8 mg of extract solubilized in 1 mL of methanol-water (1:1). Chromatographic profiles were acquired in the wavelength range from 200 to 600 nm and recorded at 280 and 320 nm. Data treatment was performed with Unipoint^®^ 2.10 software (Gilson^®^).

### 4.6. DPPH Assay 

Antioxidant activity was evaluated by the DPPH assay as described by Blois [38]. Firstly, a stock solution (2 mg extract/mL ethanol) was prepared. From this solution several dilutions were carried out. An aliquot (100 µL) of each dilution was added to the reaction medium to reach concentrations between 3.33 µg/mL and 66.67 µg/mL Subsequently, 1 mL of acetate buffer at pH 6.0, 1.4 mL of ethanol and 500 µL of DPPH (500 µM in ethanol) were added. The solutions were stirred for 30 s and afterwards left for 30 min in the dark. Absorbance at 517 nm was measured against a blank using a UV-visible spectrophotometer (Cintra 101). A control solution containing 500 µL of DPPH (500 µM in ethanol), 1 mL of acetate buffer at pH 6.0 and 1.5 mL ethanol was used. The extract antioxidant activity was evaluated from the EC_50_ value. The analysis was performed in triplicate. For formulations submitted to gastric juice, antioxidant activity was monitored through the DPPH reduction percentage, which was calculated by the equation:

(2) $$\%\;\mathrm{reduction}\;=\frac{\mathrm{Abs}\;\mathrm{control}-\mathrm{Abs}\;\mathrm{assay}}{\mathrm{Abs}\;\mathrm{control}}\times 100$$

### 4.7. Folin–Ciocalteu Method 

Total phenols were assessed with the Folin–Ciocalteu method [39]. Three aliquots of sample (100 µL) in 70% aqueous acetone were mixed with 1.9 mL of Folin–Ciocalteu reagent and, after 1 min of stirring, 5 mL of sodium carbonate solution (20%) and enough water to make 10 mL were added and stirred for 1 min. After 20 min in dark, the absorbance was measured at 700 and 735 nm against the blank. A calibration curve was obtained under the same assay conditions, using gallic acid as standard in a concentration range between 0.24 and 0.60 µg/mL. The equation of the calibration curve was used to determine total phenols, which was expressed in gallic acid.

### 4.8. Gas Chromatography (GC)

The presence of *n*-hexane traces, which was used to remove less polar compounds of the extract, was analysed by a gas chromatograph coupled to a flame ionization detector (FID) and QP2010 Plus mass spectrometer (MS; Shimadzu), comprised of a mass detector, SPL 2010, FID 2010 detector and auto sampler and a Headspace AOC-5000. The analysis was carried out by headspace, using 330 mg of extract at 80 °C for 10 min. Afterwards, 2.5 mL of the volatile phase of the sample was injected into the GC column with the following temperature program: 8 min at 50 °C, followed by an increase in the temperature at a rate of 6 °C/min up to 240 °C for 32 min, then it was kept constant 20 min.

### 4.9. Pre-Formulation Tests

#### 4.9.1. Differential Scanning Calorimetry

The analyses were performed on a heat flow Pyris 6 model DSC system (Perkin Elmer). About 2 mg of either extract or excipient, or 4 mg of the extract–excipient mixture (1:1, *w*/*w*), was analysed in a sealed aluminium pan under nitrogen flow (20 mL/min) at a heating rate of 10 °C/min, in the range 25–300 °C, with an empty, sealed pan used as reference. The equipment was calibrated with indium (99.98%, MP: 156.65).

#### 4.9.2. Infrared Spectroscopy

About 2 mg of either extract or excipient, or 4 mg of extract–excipient mixture (1:1, *w*/*w*), was analysed in a Perkin Elmer Spectrum 400 Fourier-transform infrared spectrophotometer (Perkin Elmer) through attenuated total reflectance (ATR). The sample was placed on the diamond crystal and subjected to a compression force of 100 N; spectra were obtained at 650–4000 cm^−1^, with a total of 16 scans, using a scan speed of 0.5 cm/s and a resolution of 2 cm^−1^.

### 4.10. Preparation of Capsules

Firstly, the apparent volume was determined with the procedure described in the European Pharmacopoeia (2015). The bulk density was determined by the ratio between the mass and the respective apparent volume and expressed in g/mL, which made it possible to select the number of capsules used.

Capsule contents (Table 3) were sieved (250 µm sieve) and mixed. The capsules (Capsugel/Labialpharma) were prepared with the manual filling method.

### 4.11. Quality Control of Oral Dosage Forms

#### 4.11.1. Mass Uniformity

Randomly, twenty gelatine capsules were chosen. Each one was weighed, opened and all its content was removed and weighed. The empty shell was also weighed. The mass content was determined by the difference between the two values.

#### 4.11.2. Content Uniformity

Ten capsules were randomly selected and the total phenols were quantified with the Folin–Ciocalteu method as described in Section 4.7.

#### 4.11.3. Dissolution Test

Dissolution tests of gelatine capsules were performed in a Sotax dissolution apparatus comprising a dissolution testing apparatus with an AT7 Smart paddle, a C613 Sotax fraction collector, a model CY7-50 piston pump, a continuous flow dissolution test apparatus and a computer with the program “Winsotax software off-line”. In the dissolution vessel 900 mL of artificial gastric juice were added, prepared as described in the European Pharmacopoeia [21]. For 1 L of gastric juice, 2 g of sodium chloride and 3.2 g pepsin were dissolved in water; then, 80 mL of 1 M hydrochloric acid was added and the volume was completed with water. During the assay, at 37 °C ± 0.5 °C, the stirring speed was 100 rpm, the absorbance being continuously evaluated at 333 nm in a Lambda 25 (Perkin Elmer) for 180 min. The absorbance values were reported as the rutin concentration using the following calibration curve: y = 2.0359x + 0.0007.

The dissolution test was previously validated by evaluating the specificity, linearity, accuracy and repeatability.

(a) *Specificity*—solutions of the formulation excipients (Table 3) were prepared in gastric juice and the correspondent absorbance was read at 333 nm against a blank of gastric juice;
(b) *Linearity*—a stock solution of extract in gastric juice was prepared, and from it, solutions with different concentrations of extract (100%, 80% and 60% in gastric juice) and the respective excipients of each formulation were prepared. The absorbance was read at 333 nm and the values were reported as the rutin concentration using a calibration curve;
(c) *Accuracy*—solutions with mixtures of excipients and extract (100, 80 and 60%) were prepared for the different formulations and the respective absorbance was read. The recovery percentage was calculated by the equation:
  (3) $$\%\mathrm{recovery}\;=\frac{\left[\mathrm{experimental}\right]}{\left[\mathrm{theoretical}\right]}\times 100$$
(d) *Repeatability* was evaluated by the coefficient of variation (CV) from the results of dissolution test for six capsules of each formulation, according to test conditions described above.

### 4.12. Chemical Stability of the Extract and Formulation in Artificial Gastric Juice

Polyphenol composition of the *Cymbopogon citratus* extract and of the selected formulation was evaluated after 2 and 3 h of contact with simulated gastric juice (900 mL at 37 °C). Aliquots of 1 mL were collected and purified according to Costa et al. [40]. Thus, the aliquots were placed on an Oasis^®^ HLB Cartridge column (30 mg, 1 mL; Waters^®^, Milford, MA, USA), previously activated with acidified methanol by formic acid (99.5:0.5, *v*/*v*—1 mL) and 0.5% aqueous formic acid (1 mL). After the aliquot application, 2 mL of 0.5% aqueous formic acid was added and, subsequently, polyphenols were eluted with 1.5 mL of the acidified methanol. This was dried in a rotary evaporator and the residue was taken up in 200 µL of 50% aqueous methanol. HPLC-PDA, with the conditions described in Section 4.5, was used to identify and quantify the polyphenols.

### 4.13. Antioxidant Activity of the Extract and Formulation in Artificial Gastric Juice

The antioxidant activities of the extract and formulation in simulated gastric juice at 37 °C were assessed with the DPPH assay in a similar form to that described above (Section 4.6). The samples of the dissolution medium (200 µL) were collected at time 0 and at 20 min intervals over 3 h. Aliquots (200 µL) of gastric juice at 37 °C were added to the DPPH control solution to eliminate the interference of the gastric juice and temperature in the reaction. Subsequently, the final volume was adjusted to 3 mL with ethanol. The antioxidant activity was expressed by the reduction percentage of the DPPH. The assays were performed in triplicate.

## 5. Conclusions

In the present study, it was possible to again verify that essential oil-free infusions from *Cymbopogon citratus* leaves contain a significant phenolic compound content, particularly phenolic acids, *O*- and *C*-glycosylflavones and tannins. The infusion demonstrated the capacity to reduce the DPPH radical, which indicates its ability to act as an antioxidant. The gelatin capsules developed in this work contained the extract at a similar dosage to that used in traditional medicine, and the excipients selected by the formulation tests released all their content at the after 15–20 min in gastric juice and in accordance with the quality control parameters specified in the European Pharmacopoeia for this type of formulation. The corn starch was the excipient that produced the best dissolution profile for the gelatin capsules, and it provided a promising herbal capsule formulation. Compared to unformulated extract, less degradation of phenolic compounds in gastric juice was observed with the corn starch, which indicates the protection of the extract by this excipient. Additionally, the antioxidant activity did not undergo significant changes with this formulation and in contact with gastric juice.

Thus, it can be concluded that it is possible to prepare capsules for oral administration with the *Cymbopogon citratus* extract and maintain its antioxidant activity. Additionally, the optimized formulation seems to have added value in terms of the behaviour of the phenolic compounds in gastric conditions.

Validation of in vivo antioxidant activity should be carried out in the future by high-performance liquid chromatography mass spectrometry (HPLC-MSn), in accordance with Sambiagio et al. [41], which would make it possible to quantify the isoprostane levels (the gold standard of oxidative stress) in urinary samples from individuals to whom capsules have been administered.

## Figures and Tables

**Table 1:** Identification of phenolic compounds of *Cymbopogon citratus* extract (recorded at 280 nm).

Peak	Compound *	Retention Time (min)	λ Max HPLC-PDA (nm)
1	Condensed tannin	16.78	274
2	Neochlorogenic acid	18.26	299sh;327
3	Caffeic acid derivative	19.48	298;326
4	*p*-Coumaric acid derivative	22.42	310
5	6-*C*-β-Glucopyranosyl-8-*C*-α-arabinopyranosyl luteolin	23.17	260sh;271;348
6	6-*C*-α-Arabinopyranosyl-8-*C*-β-glucopiranosyl apigenin	24.74	270;332
7	6-*C*-Pentosyl-8-*C*-hexosyl apigenin	26.10	272;338
8	6-*C*-β-Glucopyranosyl luteolin (isoorientin) 2″-*O*-β-Rhamnosyl isoorientin	27.76	258sh;270;351
9	6-*C*-Pentosyl-8-*C*-pentosyl luteolin	29.19	259sh;271;351
10	7-*O*-β-Glucopyranosyl luteolin	32.16	258sh;268;346
11	7-*O*-Neohesperosyl luteolin 6-*C*-Pentosyl-8-*C*-deoxyhexosyl luteolin	33.13	258sh;266;348
12	6-*C*-Pentosyl luteolin 2″-*O*-*α*-L-Rhamnosyl-6-*C*-α-arabinofuranosyl luteolin	35.82	258sh;270;347
13	2″-*O*-Rhamnosyl-(6-deoxy-ribo-hexos-3-ulosyl) luteolin	36.89	259sh;270;351

* Assignment made according to Figueirinha et al. [10]; Francisco et al. [22].

**Table 2:** Content uniformity of the formulations.

Figure	Theoretical Content (mg Gallic Acid) *	Experimental Content (mg Gallic Acid) **	Maximum (mg Gallic Acid)	Minimum (mg Gallic Acid)
F1	23.17 ± 0.02	24.78 ± 0.24	24.93	24.44
F2	23.17 ± 0.02	25.65 ± 0.24	25.88	25.31
F3	23.17 ± 0.02	24.33 ± 0.20	24.55	24.09

* Average ± standard deviation; ** average ± standard deviation (*n* = 10). F1—extract and corn starch; F2—extract and lactose monohydrate; F3—extract and mixture of all tested excipients.

**Table 3:** Composition of the formulations.

Excipient	Formulation (mg)			
	F1	F2	F3	F4
Extract	330	330	330	330
Corn starch	158	-	49.30	-
D (+)-Lactose monohydrate	-	199	66.50	-
Magnesium stearate	-	-	2.43	-
Microcrystalline cellulose	-	-	42.50	-

## Data Availability

Not applicable.

## Sample Availability

Sample of the extract is available from the authors.

## Abbreviations

DPPH, 2,2-diphenyl-1-picrylhydrazyl; DSC, differential scanning calorimetry; FID, flame ionization detector; GC, gas chromatography; HPLC-PDA, high performance liquid chromatography with photodiode array detector; FTIR-ATR, infrared spectroscopy with attenuated total reflection; min, minutes; mL, millilitre; MP, melting point; MS, mass spectrometer; ROS, reactive oxygen species; UV, ultraviolet.
